# Improving Inertial Sensor-Based Activity Recognition in Neurological Populations

**DOI:** 10.3390/s22249891

**Published:** 2022-12-15

**Authors:** Yunus Celik, M. Fatih Aslan, Kadir Sabanci, Sam Stuart, Wai Lok Woo, Alan Godfrey

**Affiliations:** 1 Department of Computer and Information Sciences, Northumbria University, Newcastle upon Tyne NE1 8ST, UK; 2 Department of Electrical and Electronics Engineering, Karamanoglu Mehmetbey University, Karaman 70100, Turkey; 3 Department of Sport, Exercise and Rehabilitation, Northumbria University, Newcastle upon Tyne NE1 8ST, UK

**Keywords:** human activity recognition, inertial measurement units, data augmentation, convolutional neural networks

## Abstract

Inertial sensor-based human activity recognition (HAR) has a range of healthcare applications as it can indicate the overall health status or functional capabilities of people with impaired mobility. Typically, artificial intelligence models achieve high recognition accuracies when trained with rich and diverse inertial datasets. However, obtaining such datasets may not be feasible in neurological populations due to, e.g., impaired patient mobility to perform many daily activities. This study proposes a novel framework to overcome the challenge of creating rich and diverse datasets for HAR in neurological populations. The framework produces images from numerical inertial time-series data (initial state) and then artificially augments the number of produced images (enhanced state) to achieve a larger dataset. Here, we used convolutional neural network (CNN) architectures by utilizing image input. In addition, CNN enables transfer learning which enables limited datasets to benefit from models that are trained with big data. Initially, two benchmarked public datasets were used to verify the framework. Afterward, the approach was tested in limited local datasets of healthy subjects (HS), Parkinson’s disease (PD) population, and stroke survivors (SS) to further investigate validity. The experimental results show that when data augmentation is applied, recognition accuracies have been increased in HS, SS, and PD by 25.6%, 21.4%, and 5.8%, respectively, compared to the no data augmentation state. In addition, data augmentation contributes to better detection of stair ascent and stair descent by 39.1% and 18.0%, respectively, in limited local datasets. Findings also suggest that CNN architectures that have a small number of deep layers can achieve high accuracy. The implication of this study has the potential to reduce the burden on participants and researchers where limited datasets are accrued.

## 1. Introduction

Human activity recognition (HAR, also termed activity pattern/classification) investigates objective detection of daily activities such as level walking or stair ascent [1,2,3]. HAR in neurological populations to identify periods of activity is important as it enables clinicians to better understand patients’ functional abilities, which may inform treatment or prognosis [4]. More broadly, HAR has previously been adopted in healthcare applications such as mobility and fall detection in older adults [5], adolescents with cerebral palsy [6], and stroke survivors (SS) [7] to better understand the quality of life-related outcomes.

Camera and radar-based technologies are utilized in HAR applications but are limited due to high cost, privacy issues, and computational requirements [1,8,9]. Alternatively, low-cost and lightweight wearable inertial measurement units (IMUs: accelerometer and gyroscopes) enable researchers to cost-effectively quantify longitudinal mobility data in controlled and/or free-living environments (e.g., home) [3]. Wearable IMUs [1,8,10,11] (occasionally integrated with other sensing modalities, e.g., magnetometer [12], electrocardiograph [13,14], and electromyography [15]) with contemporary classification architectures [10,11] provide highly accurate HAR. Typically, accelerometers are the dominant inertial sensor for HAR, but the inclusion of a gyroscope increases recognition accuracies by providing data from rotational activities of the trunk or legs, such as during turning, stair ascent, and descent [3]. Often, labeling wearable-based HAR data in clinics is performed manually because of the controlled conditions [16], i.e., clearly defined (scripted) periods of walking with time stamps. Moving beyond the lab creates challenges that greatly impact the practicality and use of manual segmentation, such as vast amounts of unlabeled data [11]. Consequently, artificial intelligence (AI) such as deep learning (DL) based approaches have become key to automatically identifying daily/habitual activities [17,18], fall detection [19], and negating time-consuming manual segmentation and data labeling.

Performances of automated HAR approach depend on the complexity level of the recognition process and the predictive capacity of the AI recognition models adopted since each individual tends to perform activities in different ways due to, e.g., habits, personal preferences, age, and health [3]. Studies show that wearable IMUs attached to people with neurological conditions generate different acceleration and angular velocity signals than healthy controls [20], and having such diverse data cause intra-class variations which impact model performance [21,22]. Previously, models that were trained with data belonging to healthy participants demonstrated significant drops in HAR accuracy when classifying activities of Stroke survivors (SS) [23] and people with Parkinson’s disease (PD) [24]. In order to overcome such limitations, previous studies suggested generating HAR models for each user profile [3], e.g., training a model specifically for SS and those with PD. However, accurate HAR of daily activities requires a diverse and balanced dataset [8]. Previous studies reported that existing public datasets have either a limited number of activities and participants or include data belonging to limited user profiles or limited and unbalanced data of neurological populations [3,8]. This can be attributed to: participants having difficulty performing certain daily activities due to poor mobility, challenges of data collection in healthcare due to privacy issues [10] and/or; establishment of multidisciplinary teams to aid patient/participant recruitment that are well characterized i.e., with clinical notes/records.

In this paper, we propose a methodology to investigate how limited data can be better utilized to achieve accurate HAR/mobility classification in limited healthy, PD, and SS population-specific models. To the authors’ best knowledge, this is the first study that aims to solve low HAR performances in limited datasets of neurological populations. To achieve our goal, we propose numerical-to-image conversion as the fundamental component within our proposed methodology. The use of data augmentation complements our framework by providing solutions to the limited dataset and overfitting problems. Finally, using transfer learning enable applications with small data to benefit from models that are more experienced and trained with big data. An investigation of the proposed method’s performance was initially performed on two public datasets. Results were compared to the reference studies with and without data augmentation operations in the same datasets. Then, several pilot studies tested our numerical-to-image conversion approach along with a data augmentation technique on limited local datasets belonging to healthy, PD, and SS participants. Therefore, the contributions of this study are:

1. Developing a novel framework that converts inertial sensor time-series data into images (activity images).
2. Adopting established data augmentation techniques in image processing to artificially increase limited datasets for the purpose of better HAR in neurological populations (where access to data may be difficult).
3. Verifying the proposed approach in public datasets and conducting experimental pilot studies for a single sensor-based HAR on limited HS, PD, and SS datasets.

## 2. Related Works

Machine learning (ML) algorithms, such as Support Vector Machine (SVM) or Decision Tree (DT), rely on manual feature extraction and selection that greatly impact HAR accuracy. Prior works have shown that designing hand-crafted features in a specific application requires human-based domain knowledge [25], and heuristically-defined features may perform well in recognizing one activity but not others [26]. Furthermore, hand-crafted features may not be sensitive to targeted cohorts and environments [27], i.e., models developed with a set of features in a lab lose accuracy when applied in freeliving (beyond the lab) due to the diversity of user’s habitual behavior and complexity of activities and environments. Equally, human expertise may not always select the best features, which can decrease accuracy and make it necessary to apply additional feature selection methods to reduce dimensionality [3]. The use of ensemble classifiers has been recommended to increase classification accuracy [28,29], but studies utilized complex methods that were computationally inefficient. In order to optimize performance, IMU-based HAR approaches have generally converged on DL [8]. DL algorithms are capable of generating complex and high-level features that well represent raw data and do not require expert knowledge for feature extraction and selection [3,30]. DL methods are considered state-of-the-art in computational processing [31] and have provided very accurate classification approaches [2,22].

Common DL approaches include Convolutional Neural Networks (CNN), which are able to learn multiple layers of feature hierarchies to provide high accuracy for the recognition of repetitive activities with a long duration [8]. Compared to other AI methods, CNNs have a local dependency, an ability to identify the correlation between close signals and scale invariance with an ability to work with different frequencies in time series data [2]. CNN models have been used with other AI methods, such as Long-short-term memory (LSTM) recurrent neural networks, to capture time dependencies on features extracted by convolution operations. This kind of combined architecture outperformed other studies that used the same HAR dataset [32]. Additionally, spectrogram-based feature extraction methods using Short-Time Fourier transform (STFT) from raw IMU data have been proposed through data augmentation with down sampling and shuffling techniques before classification with LSTM [33].

In both ML and DL models, the variety and size of data have the utmost importance in minimizing overfitting. Failing to provide a diverse and large data set will cause training and validation errors. Data augmentation is a powerful method to solve training, validation errors, overfitting [34,35], and data sparsity problems. Previously, a two-stage end-to-end CNN model was proposed along with an augmentation technique to enhance datasets by inserting data points via linear interpolation [36]. The results of the proposed methodology outperformed previous studies in terms of classifying activities in a dataset of healthy participants. Another study used two different time series data augmentation techniques to investigate the impact on accuracy and reported that the use of data augmentation significantly enhances recognition accuracy in three public datasets of healthy participants [37]. Alternatively, the Generative Adversarial Network (GAN) framework [38] was adopted to generate more data samples. Although GAN could improve the performance of classifiers with limited labeled data, weaknesses such as lack of explicit representation of the generator’s distribution and the need for model synchronization were reported [10]. Synthetic Minority Over-sampling technique (SMOTE) is another technique that uses oversampling to generate more data samples [39] and achieves better classifier performances in ML classifiers (such as Naive Bayes) but has not been fully investigated in DL classifiers and HAR of neurological populations.

Interpretation of numeric IMU data as images has been implemented in very few HAR studies. In [5], IMU data was stacked row by row into an array (called a signal image) before a 2D Discrete Fourier transform (DFT) was applied to generate activity images which were then input to a CNN. Elsewhere, frequency (activity) images were created from the raw IMU signals by applying STFT [22] and Fast Fourier Transform (FFT) [40] before being used as input to a CNN. However, the referenced studies performed HAR using activity images (spectrum) rather than a direct representation of numerical sensor values. Although these studies produced accurate HAR, the images (spectrum) used do not fully represent raw sensor data. Using raw sensor data to create images where pixel brightness increases/decreases with the numerical value of the IMU is a novel and potentially more accurate alternative as it better represents raw (sample level) IMU data. Previously, images that were created with this approach provided very promising classification results of the survival status of the patient using a clinical record dataset [41].

### Inertial Sensor-Based HAR in Neurologic Populations

The use of inertial sensors in HAR eliminates immediate privacy and security concerns and offers pragmatic data collection possibilities via various technologies such as commercially available devices, smartphones, and smartwatches. Despite providing unique opportunities, inertial sensor-based HAR also poses many challenges, such as accurately recognizing the activity type from an unknown environment using an inertial signal [1]. Unlike camera-based HAR systems, inertial sensor-based HAR requires additional mechanisms such as video recording or scripted data collection protocol to label the data before training. Another challenge posed by inertial sensor-based HAR is the requirement of wearing multiple sensors. Although multiple inertial sensors-based HAR has provided highly accurate activity classification [22], wearing multiple devices may cause discomfort while increasing computation and project costs. Accordingly, most studies utilize a single waist-mounted sensor [42].

Several publicly available benchmark datasets have been generated using a single sensor configuration to enable researchers to develop highly accurate HAR models [43,44]. However, those datasets were produced from healthy people only [2]. The lack of HAR benchmarking datasets for neurological populations force researchers to create local (project-specific) datasets. The creation of a local dataset that has diverse and sufficient data is challenging due to several reasons [10]. For example, researchers interested in HAR within neurological disorders may struggle with patient recruitment (due to a lack of clinical partners) or ensure the longevity of recording to obtain sufficient data due to a lack of patient adherence. Additionally, data may be skewed as those with functional limitations may generally perform light activities only, such as level ground walking rather than stair ascent/descent or walking over uneven terrain due to fear of falling. These real-life implications result in datasets of SS [27,45], PD [24], and people with spinal cord injury [46] that may not be rich and diverse enough to achieve very high HAR accuracies on new data.

Accurate HAR in neurological populations requires diverse data from multiple participants with a broad range of ages, fitness levels, disease duration, mood, and health conditions to ensure inter-subject and intrasubject variability have minimal impact on recognition accuracy [47]. For example, people with different stroke types (e.g., ischemic, hemorrhagic) and post-stroke recovery durations may show different levels of impaired mobility during stair ascending/descending. Increasing the size of the dataset may also contribute to minimizing the impact of subject variability in classification models.

In this study, we hypothesize that converting numerical sensor data into activity images and implementing data augmentation techniques can alleviate diversity and data balance issues, thereby increasing the performance of DL methods by utilizing well-established techniques in image processing [48]. The use of image data for training and testing models makes CNN models a viable choice because CNN models not only extract high-level features from images but also present more compactly and robustly what the image essentially represents.

## 3. Methodology

**Figure 1:**
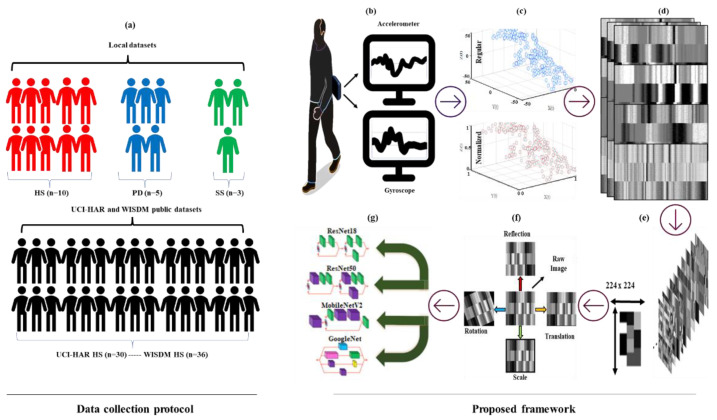
Data collection protocol and proposed framework: (**a**) Dataset illustration. The flow of the proposed HAR methodology with data augmentation and CNN architectures: (**b**) IMU data acquisition, (**c**) data normalization, (**d**) numerical to image conversion, (**e**) resizing, (**f**) data augmentation, (**g**) CNN classification.

The proposed methodology developed for better HAR of people with neurological conditions is presented in Figure 1. Three limited local datasets and two independent benchmarking public datasets were used to verify the proposed methodology. In order to replicate the pragmatic problems in this domain, the local dataset has a limited number of participants, data sparsity, and class imbalance. In the proposed methodology, numerical inertial sensor data were first normalized and then converted into images (initial state). Then, established image augmentation techniques were adopted to artificially increase the number of images (enhanced state). Finally, generated images were fed into different CNN architectures. All steps are further detailed in this section.

### 3.1. Data Normalization and Numerical to Image Conversion (Initial State)

Raw accelerometer and gyroscope signals experience different lower and upper limits because of configuration (e.g., an accelerometer typically can collect data in the range of ±16 m/s^2^, whereas gyroscopes can sense up to ±2000°/s). Normalizing features with different upper and lower limits is a commonly used pre-process in AI, as extreme differences between different features may have a negative impact on learning abilities [49]. In the normalization step, a feature scaling-based normalization method is preferred due to its convenience. Here, raw IMU data (x) is normalized ($\hat{x}$) considering max value ($x_{max}$) and min value ($x_{min}$), as depicted in Figure 1c. As a result of normalization, the value in matrices ranges between 0 and 1 for both accelerometer and angular velocity, Equation (1)

(1) $$\hat{x}=\frac{x-x_{min}}{x_{max}-x_{min}}$$

After normalization, data were divided into sub-segments (windows), considering each sub-segment should contain sufficient characteristics that allow HAR to be successfully performed. A previous study [50] investigated windows size impact on HAR application and reported that the ideal size for fixed windows ranges between 2 s and 5 s considering a frequency of 20 Hertz (Hz) to 50 Hz. Therefore, each activity was divided into consecutive segments of fixed-length (≈2.5 s windows), considering that at least two strides are needed to recognize walking and stair ambulation. IMUs typically sense tri-axial acceleration ($a_{x},a_{y},a_{z}$) and tri-axial angular velocity ($w_{x},w_{y},w_{z}$) in the t moment (Equation (2)). Generally, popular CNN models are not suitable to use 1D datasets and require 2/3D images to feed input layers [51]. Therefore, many previous studies [32,52,53] extract IMU data features with 1D convolution layers and then evaluate those features with recurrent neural network-based methods. Here, we convert numerical IMU data to images to go beyond that limit, as shown in Figure 1d.

(2) $${\mathrm{IMU}}_{t}=\left[a_{x}{}_{{}_{t}}{,a}_{y}{}_{{}_{t}}{,a}_{z}{}_{{}_{t}}{,w}_{x}{}_{{}_{t}}{,w}_{y}{}_{{}_{t}}{,w}_{z}{}_{{}_{t}}\right]$$

Equation (3) represents 2D data (also can be referred to as an image) created by vertical placement of accelerometer and gyroscope values recorded in 2.50 s window/250 sample and 2.56 s windows/128 sample for the local dataset and UCI HAR dataset, respectively. In the WISDM dataset, only accelerometer values were placed in a 2.50 s window/50 sample. Unlike previous studies [5,22,40], this study ensures that each numerical IMU value corresponds to a specific pixel in an image. The normalized values in the matrices were multiplied by 255 to produce grey images with pixels ranging from 0 to 255. As a result, images whose brightness increases/decreases with the numerical value of the IMU are produced. However, image dimensions are not suitable to feed the input layer of CNN models since each CNN model’s input layer accepts images with a size of 224 × 224 [51]. Therefore, resizing is applied by stretching the row length to obtain a square matrix from these images Figure 1e.

(3) $$\left[\begin{array}{l} {\mathrm{IMU}}_{{\mathrm{acc}}_{x}}\\ {\mathrm{IMU}}_{{\mathrm{acc}}_{y}}\\ {\mathrm{IMU}}_{{\mathrm{acc}}_{z}}\\ {\mathrm{IMU}}_{{\mathrm{gyro}}_{x}}\\ {\mathrm{IMU}}_{{\mathrm{gyro}}_{y}}\\ {\mathrm{IMU}}_{{\mathrm{gyro}}_{z}} \end{array}\right]=\left[\begin{array}{l} a_{x_{t}}{,a}_{x_{t+1}}{,a}_{x_{t+2}},\ldots \ldots {,a}_{x_{t+126/248\;}}{,a}_{x_{t+127/249}}\\ a_{y_{t}}{,a}_{y_{t+1}}{,a}_{y_{t+2}},\ldots \ldots {,a}_{y_{{}_{t+126/248\;}}}{,a}_{y_{t+127/249}}\\ a_{z_{t}}{,a}_{z_{t+1}}{,a}_{z_{t+2}},\ldots \ldots {,a}_{z_{t+126/248\;}}{,a}_{z_{t+127/249}}\\ w_{x_{t}}{,w}_{x_{t+1}}{,w}_{x_{t+2}},\ldots \ldots {,w}_{x_{t+126/248\;}}{,w}_{x_{t+127/249}}\\ w_{y_{t}}{,w}_{y_{t+1}}{,w}_{y_{t+2}},\ldots \ldots {,w}_{y_{{}_{t+126/248\;}}}{,w}_{y_{t+127/249}}\\ w_{z_{t}}{,w}_{z_{t+1}}{,w}_{z_{t+2}},\ldots \ldots {,w}_{z_{{}_{t+126/248\;}}}{,w}_{z_{t+127/249}} \end{array}\right]$$

### 3.2. Data Augmentation

Table 1 presents the number of occurrences along with class distribution in limited local datasets. In order to alleviate the problems related to small dataset size and prevent overfitting; data augmentation was applied to increase the number of generated images using established image processing techniques. In this sense, four different image position augmentation techniques (reflection, rotation, scale, and translation) were applied to each image to ensure data diversity and robust training, see Figure 1f. Reflection, also known as symmetry, is an image pre-processing operation that can occur in horizontal or vertical access. Rotation, scaling, and translation is other pre-processing operations that deal with spinning, resizing, and moving (right, left, up, and down) in given upper and lower limits, respectively. The lower and upper limit values of rotation, translation (pixel), and scale are ±30°, ±10°, and 0.9–1.1, respectively, since these values have proved to be efficient [41]. Consequently, the size of the original dataset in the initial state was enhanced by adding 8 times more artificial data (4 different techniques with lower and upper limits). In this context, the number of occurrences for each class in the local datasets is increased, Table 2.

### 3.3. HAR via CNN

Benchmarking analysis of various deep learning models was previously studied, and performance indices such as accuracy, model complexity, memory usage, computing power, and interference times were evaluated [51,54]. We determined our priority performance indices as high accuracy rate, minimal computing power, and short prediction time to achieve an effective HAR framework. Therefore, we chose four optimal pre-trained networks GoogleNet [55], ResNet18 [56], ResNet50 [56], and MobileNet-v2 [57,58], in the Pareto frontier as these architectures satisfy our requirements. Each CNN architecture used in this study differs from the others in layer, size, and parameters and is often preferred in benchmarking studies to evaluate CNN performances [59,60], Table 3. MATLAB^®^ (2021, MathWorks, Inc., Natick, MA, USA) software on a laptop with Intel Core i7-7700HG CPU (2.80 GHz), 16 GB RAM, NVIDIA GeForce GTX 1050 4 GB was used to perform CNN training and testing.

A residual network (ResNet) [56] was developed to improve unexpected low performances of deeper network architectures by adding a skip connection (shortcut) to convey information between layers and avoid the vanishing gradient problem [60]. There are different ResNet variants (18-layer, 34-layer, 50-layer, 101-layer, 152-layer) proposed considering the number of layers and output sizes. ResNet18 and ResNet50 were implemented here. MobileNet was employed as it has low computation and fast operation by using depth-wise separable convolutions to reduce the number of parameters and computation time. Specifically, MobileNet-v2 [58] was implemented, which has 54-layers, distinguishing it from MobileNet in using inverted residual blocks with bottleneck properties. GoogleNet [55] is 22-layer deep (excluding pooling) model designed with computational efficiency and practicality. It uses the inception module to extract features more effectively using various filter sizes. And the computational load is reduced with a 1 × 1 convolution of the depth of the network. Minor adjustments, such as the use of fine-tuning networks were made to the existing architecture for the four-class classification problem in this study. In this context, a fully connected layer with four outputs and a classification layer was added to the existing structure, see Figure 1g.

## 4. Datasets

### 4.1. Local Datasets

Ten HS (28.4 ± 7.0 years, 79.2 ± 14.4 kg, 176.8 ± 8.4 cm, 8 Male, M: 2 Female, F), five people with PD (61.5± 3.43 years, 82.9 ± 10.3 kg, 175.8 ± 4.6 cm, 5M) and three SS (72.3 ± 3.1 years, 78.5 ± 12.1 kg, 176 ± 8.2 cm, 3M) were recruited, as illustrated in Figure 1a. Each participant was instructed to stand for 2-min (eyes open and comfortable standing), then walk over the level ground for 2-min around a 20-m circuit at their self-selected walking speed inside the lab. Afterward, participants ascended and descended stairs (15 steps) outside of the lab (in a generic university campus stairwell).

Assessment and instrumentation were carried out by a physiotherapist and a trained researcher, respectively. Ethical consent was granted by the Northumbria University Research Ethics Committee (REF: 21603). All participants gave informed written consent before participating in this study. Testing took place inside and outside of a gait laboratory/lab, Coach Lane Campus, Northumbria University, Newcastle, upon Tyne.

Each participant wore a Shimmer3 IMU device (5.1 cm × 3.4 cm × 1.4 cm, 23.6 g) on the 5th lumbar vertebrae (L5), as shown in Figure 1b. IMU signals (tri-axial accelerometer and tri-axial gyroscope) were recorded at a sampling frequency of 100 Hz and configured with 16-bit resolution (±8 g, ±500°/s). IMU data were transferred to a workstation (Windows 10) from the IMU device via proprietary software (Consensys, Shimmer). Labeling of activities in a continuous data stream was conducted via a wearable camera for PD and SS, whereas a scripted experimental protocol was used for HS. All participants performed the same protocol. Inertial data streams for each activity were segmented into 2.5 s (250 sample points) windows with 50% overlap using a sliding window.

### 4.2. UCI-HAR and WISDM Independent Benchmarking Datasets

UCI-HAR dataset [44] was preferred to test the development methodology as it was created using the same data collection protocol as the local dataset. UCI-HAR dataset has an accelerometer and gyroscope recording of 30 HS (19–48 years), collected by a device attached at waist level. The dataset was randomly portioned into training and testing. Data were recorded at a sampling frequency of 50 Hz and segmented to fixed-width sliding windows of 2.56 s (128 sample points) with 50% overlap. The WISDM dataset was created from 36 HS under controlled laboratory conditions. The dataset has tri-axial accelerometer readings only recorded at 20 Hz. Accelerometer recordings were segmented to fixed-width sliding windows of 2.50 s with 50% overlap.

Table 4 presents activity classes along with class distributions in the benchmarking datasets. Skewed class distributions are present in the public datasets. This typically limits the learning/training process by causing class overlapping, small sample size, or small disjuncts [61]. In addition, models trained with imbalanced datasets are often biased towards the majority class, and therefore there is a greater misclassification rate for the minority class occurrences such as sitting and standing in the WISDM dataset [62]. Furthermore, the most common evaluation metric, accuracy, treats all classes as equally important, which makes it inefficient [3]. To alleviate the limitations of imbalanced public datasets, we utilized 500 occurrences from each class for training in public datasets. In total, 3000 occurrences were utilized for each dataset, and the train/test split ratio, along with occurrence numbers, are presented in Section 5.

## 5. Analytical Procedures

This section presents the results of the classification models in the initial state (after numerical to image conversion) and enhanced state (after data augmentation). In local datasets, 80% of the data (occurrence/images) were used for training and 20% for testing. In UCI-HAR and WISDM public datasets, a total of 3000 occurrences (500 for each class) were utilized for each dataset, where 80% (2400 occurrence/images) were used for training, and 20% (600 occurrence/images) were used for testing. Five quantitative metrics are used to evaluate the performance of each model, Equations (4)–(8). Accuracy is the most common metric and gives a general representation of model performance, but it can be inefficient when used in unbalanced datasets. Accordingly, sensitivity and specificity were also calculated as additional evaluation matrices to evaluate classes separately. F1-measure deals with a score resulting from the combination of precision and recall value, where TP: true positive, TN: true negative, FP: false positive, and FN: false negative. In addition, Matthew’s correlation coefficient (MCC) was included as it includes TN, unlike F1-measure. Total execution time was also calculated for enhanced states of all models.

(4) $$\mathrm{Accuracy}=\frac{\mathrm{TP}+\mathrm{TN}}{\mathrm{TP}+\mathrm{FP}+\mathrm{TN}+\mathrm{FN}}\times 100$$

(5) $$\mathrm{Sensitivity}=\frac{\mathrm{TP}}{\mathrm{TP}+\mathrm{FN}}$$

(6) $$\mathrm{Specicificity}=\frac{\mathrm{TN}}{\mathrm{TN}+\mathrm{FP}}$$

(7) $$F1-\mathrm{score}=\frac{2\times \mathrm{TP}}{2\times \mathrm{TP}+\mathrm{FP}+\mathrm{FN}}$$

(8) $$\mathrm{Matthew}’s\;\mathrm{correlation}\;\mathrm{coefficient}\left(\mathrm{MCC}\right)=\frac{\left(\mathrm{TP}\times \mathrm{TN})-(\mathrm{FP}\times \mathrm{FN}\right)}{\sqrt{\left(\mathrm{TP}+\mathrm{FN})\times (\mathrm{TN}+\mathrm{FP})\times (\mathrm{TP}+\mathrm{FP})\times (\mathrm{TN}+\mathrm{FN}\right)}}$$

## 6. Results

### 6.1. UCI-HAR Datasets

Table 5 presents the results of performance metrics for initial and enhanced states in UCI-HAR. In the initial state, ResNet18 architecture slightly outperformed its counterparts in all performance metrics. Moreover, the data augmentation operation provided slight improvements in the performance metrics of each architecture, whereas the largest improvement was observed in GoogleNet. In the enhanced state, ResNet50 architecture provided slightly higher performances compared to other CNN architectures and reached 97% accuracy. However, comparing execution time reveals that GoogleNet classifies HAR activities faster than its counterparts. Table 6 presents the ResNet50 confusion matrix of the UCI-HAR dataset in the initial and enhanced states as it outperforms other architectures in terms of all performance metrics except execution time. Here, notable improvements are observed after data augmentation, especially in static activities (sitting, standing, and laying).

### 6.2. WISDM Datasets

Table 7 presents the classification results of the four CNN architectures using the WISDM dataset in initial and enhanced states. In the initial state, ResNet50 architecture classified HAR activities better than ResNet18 and MobileNet-v2, whereas GoogleNet showed a notably poorer performance. However, this is not valid for specificity metrics which experienced similar values in all architectures. After data augmentation is implemented, significant improvements are observed in all architectures. ResNet18 reached 95.8% accuracy with the shortest training time, whereas ResNet50 and MobileNet-v2 provided slightly lower accuracies but in a much longer time (≥130 min). Although GoogleNet is improved in its enhanced state, it is still the poorest in activity recognition compared to other architectures. Table 8 presents the confusion matrix for the best-enhanced state (ResNet18). Comparing activity recognition performances for each class in the initial and enhanced state reveals that the largest improvements are obtained in the accurate recognition of static activities (sitting and standing).

### 6.3. Local Datasets (HS Model)

Table 9 shows the initial and enhanced state results of HAR in the local dataset created from HS. In the initial state, MobileNet-v2 architecture outperforms its counterparts in terms of each performance metric, whereas GoogleNet architecture performs poorly in recognition of HAR activities. Significant improvements are observed in the enhanced state where ResNet50 reaches the highest accuracy with 100%, especially GoogleNet accuracy is more than doubled in the enhanced state. Table 10 presents the confusion matrix created from ResNet50 architecture, which experienced misclassification in recognition of stair activities in the initial state. After data augmentation, ResNet50 architecture better-adopted stair classes and corrected the misclassifications.

### 6.4. Local Datasets (PD Model)

Table 11 presents the initial and enhanced results of HAR in those with PD. In the initial state, all CNN architectures experience comparable results where ResNet18 and ResNet50 outperform other architectures. Later in the enhanced state, notable improvements were observed in all architectures, but MobileNet-v2 achieved the highest performance. Table 12 presents a confusion matrix belonging to the classification result of MobileNet-v2, where misclassification in stair descent and walking activities were improved in the enhanced state.

### 6.5. Local Datasets (SS Model)

Table 13 shows performances from initial and enhanced states in the local SS dataset. In the initial state, ResNet18, ResNet50, and MobileNet-v2 experience accuracies just above 70%, whereas GoogleNet shows the poorest performance with 65.7% accuracy. In the enhanced state, all architectures except GoogleNet experience significant improvements and reach over 95% accuracy. On the other hand, GoogleNet also experiences improvements but with a small margin compared to its counterparts. Table 14 presents the confusion matrix of ResNet50 from initial and enhanced states. In the SS group, stair ascent occurrences were mostly misclassified, whereas stair descent and walking activities suffered from low recognition. In the enhanced state, notable improvements were observed, especially in stair activities.

## 7. Discussion

The computational performance of the framework was deemed acceptable for data preparation (normalization, generally having low computational cost). Specifically, normalization of each segmented IMU window took approx. 5.4 milliseconds which was then converted into the activity image within approx. 2.1 milliseconds are, resulting in total data preparation for each occurrence of about 7.5 milliseconds. However, model training was prolonged and is discussed in Section 7.3, Limitations. Here, we first verify the proposed approach in benchmarking datasets and compare it with reference studies, Section 7.1. This tests whether the proposed numerical-to-image conversion approach is a valid and reliable approach in independent datasets. Results suggest that the proposed framework can classify activity classes in both benchmarking datasets with high accuracy, especially after data augmentation. The pre-trained networks used in this study can achieve better or comparable classification accuracies against reference studies even when the networks are trained with a portion of the original datasets.

After promising results are obtained in benchmarking datasets, we provide an evaluation regarding the pilot studies (in HS, PD, and SS), which test the proposed approach (numerical to image conversion and data augmentation) on limited local datasets. In addition, we present an analysis regarding why some CNN architectures perform better than others and recommend the necessary properties a pre-trained network needs to achieve sufficient learning.

### 7.1. Verification of the Results in Public Datasets

Table 15 compares the proposed framework against several reference studies with and without data augmentation in the same public datasets. Overall, numerical-to-image conversion, along with data augmentation, significantly improves the performance of CNN architectures in HAR. This study utilized 500 occurrences/instances for each class to provide unbiased evaluation metrics, as detailed in 4.2. Therefore, our findings should be considered in this context.

#### 7.1.1. UCI-HAR Dataset

Comparing our initial results with a reference study [37] initial results in the same dataset reveals that the proposed numerical-to-image conversion approach is an effective method. Here, ResNet18 architecture reaches 93.3 % accuracy, which is superior to 80% accuracy [37]. In the enhanced state of the UCI-HAR dataset, the methodology proposed here provides similar or better results compared to the reference studies, Table 15. Comparing the training times with a reference study [37] that uses an exponential smoothing augmentation technique reveals that our approach reaches 97.0% accuracy in 166 min training duration, whereas the reference study reaches 97.9% accuracy in 210 min. This suggests that the proposed framework can provide comparable accuracies with smaller training data with shorter durations. The difference in the training times could be attributed to the preferred data augmentation technique. For example, the exponential smoothing approach assigns exponentially decreasing weights for older observations. However, our framework uses raw numerical data to produce activity images that are independent of the numerical values in the data stream. Producing images (e.g., activity images or spectrogram) directly from raw sensor data was proved to be effective in HAR [5,22,40].

#### 7.1.2. WISDM Dataset

In the initial state, our numerical-to-image conversion technique with ResNet50 reaches 86% accuracy, which is superior to 83.4% in [37] and comparable to 86.4% in [36]. In the enhanced state, our accuracy reaches 95.8% with ResNet18 architecture, which is comparable to 95.7% in [36] but poorer than 97.1% in [37]. Comparing the training time with a reference study [37] reveals that our proposed framework reaches comparable accuracies with smaller training data and shorter training duration.

### 7.2. Verification in Local Datasets

We tested the proposed approach (initial state and enhanced state) on local datasets of HS, PD, and SS groups. In the initial state, in terms of accuracy, CNN architectures provide higher performances in the PD dataset compared to HS and SS. This could be associated with the fact that the PD dataset is more balanced than SS and larger than both HS and SS. In addition, majority classes (walking and standing) are better recognized than minority classes (ascent and descent) in the PD dataset. When the sizes of the datasets were artificially increased with data augmentation techniques in the enhanced state, improvements were achieved in all CNN architectures. It is important to highlight that data augmentation has no impact on the balance of a dataset because each class is enhanced at the same rate.

**Figure 2:**
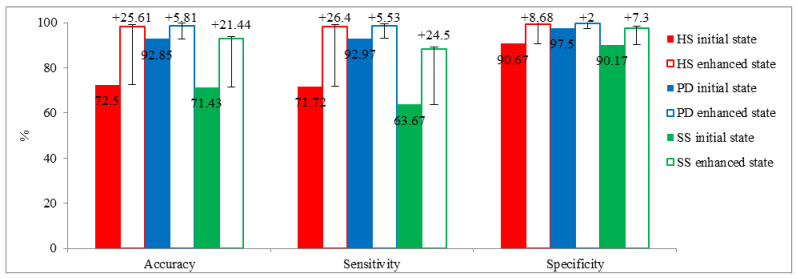
Comparison of performance matrices between initial and enhanced states in the local dataset. Sensitivity and specificity values are normalized to 0–100 to provide comparable results with accuracy.

Figure 2 presents the average performances of all CNN architectures from Table 9, Table 11, and Table 13. Sensitivity and specificity values were normalized to 0–100 to present comparable results against accuracy. Comparing initial and enhanced results considering the overall performance of all CNN architectures in the local datasets reveals that the largest improvement in terms of accuracy is observed in HS with 25.6%, followed by SS with 21.4% and PD with 5.8%, as seen in Figure 2. Comparing accuracy, sensitivity, and specificity reveals that data augmentation had the largest improvement in sensitivity at 18.81%, followed by the accuracy at 17.62% and relatively small improvements in specificity at 5.99%. This finding could be associated with the nature of the limited and imbalanced local datasets. In the initial state, the number of true positive (TP) and true negative (TN) in the classification were relatively low. After data augmentation, models experienced better performance in predicting positive classes compared to negative classes. This resulted in a larger increase in TP compared to TN. Consequently, improvements in sensitivity were found to be significantly larger than specificity, Equations (4)–(6).

**Figure 3:**
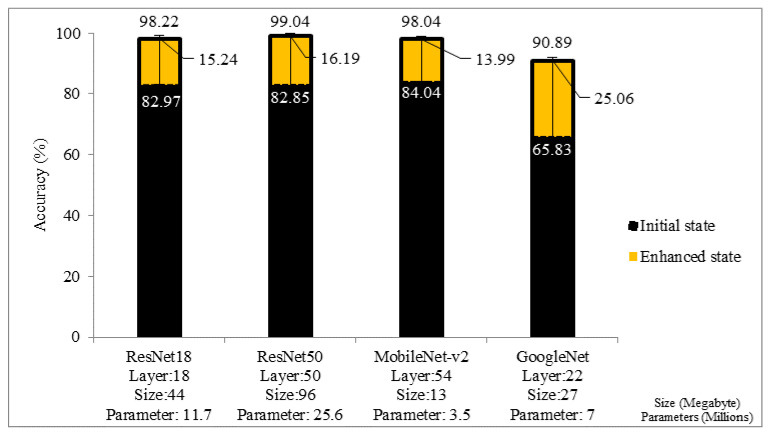
Comparison of CNN architectures in terms of accuracy in initial and enhanced status in the local datasets (HS-SS-PD combined).

All four CNN architectures showed a test accuracy exceeding 90% in the enhanced state. ResNet50 outperformed all other architectures in the enhanced state, whereas MobileNet-v2 achieved the best result in the initial state. Although GoogleNet architecture experienced the sharpest enhancement after data augmentation, overall performance in both initial and enhanced states is poorer than its counterparts, as shown in Figure 3. Interpreting these outcomes with the properties of pre-trained CNN architectures (Table 3) could provide useful information regarding the most suitable CNN architecture. Initially, comparing ResNet18 (18 layers) with ResNet50 and MobileNet-v2 (50 and 54 layers) reveals that a higher network layer does not necessarily provide better accuracy because ResNet18 achieved comparable results, aligning with the findings of a previous study that employs the same CNN architectures [60]. This suggests that network size and the number of parameters that a network can learn also have an impact on accuracy. Among the two architectures with the greatest number of deep layers, ResNet50 (larger size and more parameters) provides better classification than MobileNet-v2 (smaller size and fewer parameters) in the enhanced state. Alternatively, MobileNet-v2 (smaller size and fewer parameters) achieves better results than ResNet50 (larger size and more parameters) in the initial state where the dataset is limited and unbalanced. This phenomenon can also be partially observed when two architectures with the lowest number of deep layers are compared. ResNet18 (larger size and more parameters) achieves higher performance than GoogleNet (smaller size and fewer parameters) in the enhanced state. As a result, findings of enhanced state suggest that CNN architectures require approximately 22 deep layers and 7 million parameters (GoogleNet) to classify walking, standing, ascent, and descent activities with more than 90% accuracy. In order to achieve better accuracy, the number of deep layers and/or the number of parameters needs to be increased. The maximum accuracy can be potentially achieved with approximately 50 deep layers and 25.6 million parameters (ResNet50) or approximately 54 deep layers and 3.5 million parameters (MobileNet-v2) because ResNet50 and MobileNet-v2 were found superior in HS, SS, and PD datasets, respectively. On occasions when training time is considered as important as accuracy, ResNet18 architecture could be potentially a more suitable choice because this architecture has fewer deep layers and fewer parameters (fewer computation costs) than ResNet50. However, inconsistencies can occur, as the previous study [51] reports that not all CNN architectures use their parameters with the same level of efficiency.

**Figure 4:**
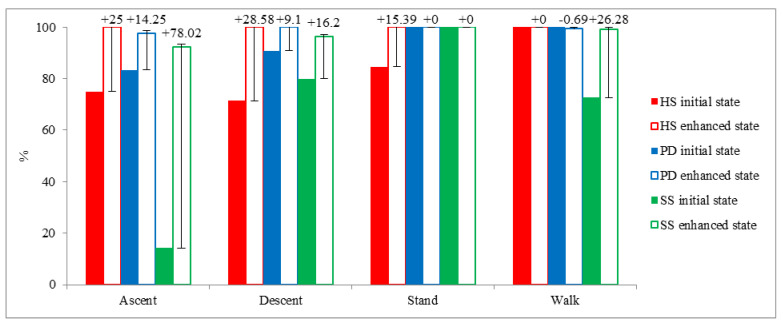
Recognition accuracy comparison of each activity in the initial result of the local dataset. This graph was derived from the architectures that provide the best performances in enhanced results.

Our findings revealed that walking and standing are recognized with higher accuracy compared to stair activities, as shown in Figure 4. We also found stair ascent is the activity with the lowest recognition accuracy, aligning with many previous studies that use a single waist device [23,36,66]. Moreover, the figure reveals that data augmentation contributes to better detection of stair ascent and stair descent by 39.1% and 18.0%, respectively. These findings align with a similar study [36] where data augmentation was shown to be effective in recognizing stair activities. Recognition of basic daily life activities in PD and stroke populations with high accuracy has the potential to provide more robust and accurate movement analysis in real life. This framework can be used to accurately classify walking bouts and assist the extraction of clinically important spatiotemporal parameters during walking. Moreover, it can also provide a better picture of the functional capabilities of people with PD and stroke by recognizing stair ambulation activities more accurately.

### 7.3. Limitation and Future Work

A limitation of the work includes total model training time. Deep learning models are structurally different from traditional machine learning models and involve significantly more training parameters, Table 3. Therefore, deep learning-based CNN models are more complex than traditional machine learning models [67]. This computational complexity can be observed in training times in Table 5 and Table 7. Although the training time reported in this study is shorter than a reference study [37], it still needs improvements.

In this study, the framework was examined within the context of four basic mobility tasks only. In addition, the dataset was created in a semi-controlled environment with a scripted experimental protocol, i.e., all participants walked in the same route while wearing the same device. Future studies will aim to investigate the performances of more complex daily activities in free-living environments (e.g., home). In addition, this framework can be deployed to advanced microcontrollers (Raspberry pi 4- 1.5 GHz) to perform real-time HAR. However, this could still be slower than offline computing as a faster CPU (Core i7-7700HG-2.80 GHz) is used in this study.

## 8. Conclusions

HAR models typically suffer from low recognition accuracy in neurological populations due to the limitations in data collection. Although highly accurate models have been developed in HAR of healthy people, these models have been found to be limited when recognizing the activities of people with walking impairments. The lack of suitable datasets for those with neurological movement disorders is a major limitation in HAR research. This study proposes a framework to enhance limited HAR datasets, which will have utility in those with a neurological movement disorder. Results showed significant improvements in HAR. The implication of this study can complement future HAR studies where the creation of diverse and balanced data sets may not be feasible. Making maximum use of limited data is important to ensure those with physical impairments may not need to perform difficult dynamic tasks for longer periods to create rich datasets. Therefore, the proposed framework also has the potential to reduce the participant and researcher burden to generate complex and diverse datasets.

## Figures and Tables

**Table 1:** Class distributions in local datasets (initial state).

Dataset	Walking	Ascent	Descent	Standing	Total
HS	50 (25)	50 (25)	49 (25)	50 (25)	199 (100)
PD	81 (29)	64 (23)	60 (21)	75 (27)	280 (100)
SS	49 (28)	18 (11)	31 (18)	75 (43)	173 (100)

The number of occurrences/images (% class distribution).

**Table 2:** Number of occurrences after data augmentation (enhanced state) in the local dataset.

Dataset	Walking	Ascent	Descent	Standing	Total
HS	450	450	441	450	1791
PD	729	576	540	675	2520
SS	441	162	279	675	1557

The number of occurrences/images (% class distribution).

**Table 3:** Properties of pre-trained CNN architectures.

CNN Architecture	Layer (Depth)	Size (Megabyte)	Parameters (Millions)	Input Image Size
ResNet18	18	44	11.7	224 × 224
ResNet50	50	96	25.6	224 × 224
MobileNetv2	54	13	3.5	224 × 224
GoogleNet	22	27	7	224 × 224

**Table 4:** Class distributions in benchmarking datasets (initial state).

Dataset		Walking	Ascent	Descent	Sitting	Standing	Laying	Jogging	Total
UCI-HAR	Original	1226 (17)	1073 (15)	986 (13)	1286 (17)	1374 (19)	1407 (19)	-	7352
	Utilized	500 (16.6)	500 (16.6)	500 (16.6)	500 (16.6)	500 (16.6)	500 (16.6)	-	3000
WISDM	Original	424,400 (38.6)	122,869 (11.2)	100,427 (9.1)	59,939 (5.5)	48,395 (4.4)	-	34,217 (31.2)	756,030
	Utilized	500 (16.6)	500 (16.6)	500 (16.6)	500 (16.6)	500 (16.6)	-	500 (16.6)	3000

Number of occurrences/images (% class distribution).

**Table 5:** HAR performance metrics in UCI HAR dataset.

		Initial State						Enhanced State				
DL-CNN Epochs: 5 Iteration: 3750 Learning rate: 0.001 Batch size: 32	Pre-trained network	Acc. (%)	Sens.	Spec.	F1	MCC	Acc. (%)	Sens.	Spec.	F1_	MCC	Training time (min)
	ResNet18	93.3	0.929	0.987	0.928	0.915	96.1	0.960	0.992	0.961	0.953	89.26
	ResNet50	91.8	0.914	0.984	0.911	0.897	97.0	0.970	0.994	0.970	0.964	165.38
	MobileNet-v2	90.7	0.903	0.982	0.899	0.883	96.2	0.962	0.992	0.962	0.954	143.41
	GoogleNet	81.0	0.803	0.962	0.800	0.771	91.9	0.919	0.984	0.918	0.903	75.55

Acc.: accuracy, Sens.: sensitivity, Spec.: specificity, F1: F1_score.

**Table 6:** Confusion matrix of UCI HAR- ResNet50 (initial results—left, final results—right).

	Walking	Ascent	Descent	Sitting	Standing	Laying		Walking	Ascent	Descent	Sitting	Standing	Laying
**Walking**	106	0	0	0	0	0	**Walking**	494	0	1	0	0	0
**Ascent**	0	108	0	0	0	0	**Ascent**	1	547	3	0	0	0
**Descent**	0	1	106	0	0	0	**Descent**	1	0	477	0	0	0
**Sitting**	0	0	0	89	3	12	**Sitting**	0	0	1	465	12	17
**Standing**	1	1	0	14	66	11	**Standing**	0	0	0	19	461	11
**Laying**	0	0	0	2	4	76	**Laying**	0	0	0	15	8	467

**Table 7:** HAR performance metrics in the WISDM dataset.

		Initial State						Enhanced State				
DL-CNN Epochs: 5 Iteration: 3750 Learning rate: 0.001 Batch size: 32	Pre-trained network	Acc. (%)	Sens.	Spec.	F1	MCC	Acc. (%)	Sens.	Spec.	F1	MCC	Training time (min)
	ResNet18	83.5	0.832	0.967	0.828	0.799	95.8	0.958	0.992	0.958	0.949	72.2
	ResNet50	86.0	0.854	0.972	0.854	0.827	95.4	0.953	0.991	0.953	0.944	163.49
	MobileNet-v2	82.7	0.821	0.965	0.821	0.787	95.4	0.953	0.991	0.953	0.944	129.52
	GoogleNet	71.5	0.719	0.943	0.718	0.678	89.3	0.891	0.979	0.892	0.871	80.27

Acc.: accuracy, Sens.: sensitivity, Spec.: specificity, F1: F1_score.

**Table 8:** Confusion matrix of WISDM dataset-ResNet18 (initial results—left, final results—right).

	Jogging	Walking	Ascent	Descent	Sitting	Standing		Jogging	Walking	Ascent	Descent	Sitting	Standing
**Jogging**	100	2	0	3	0	1	**Jogging**	488	3	3	1	0	0
**Walking**	0	106	0	2	0	0	**Walking**	0	546	0	5	0	0
**Ascent**	3	4	85	12	0	3	**Ascent**	5	6	453	8	2	4
**Descent**	3	4	8	79	5	5	**Descent**	0	6	20	457	4	8
**Sitting**	0	0	0	1	60	32	**Sitting**	0	0	3	1	468	19
**Standing**	0	1	0	0	10	71	**Standing**	0	0	4	2	21	463

**Table 9:** HAR performance in local HS dataset.

		Initial State					Enhanced State				
DL-CNN Epochs: 5 Iteration: 190 Learning rate: 0.001 Batch size: 32	Pre-trained network	Acc. (%)	Sens.	Spec.	F1	MCC	Acc. (%)	Sens.	Spec.	F1	MCC
	ResNet18	80.0	0.821	0.936	0.803	0.753	99.7	0.997	0.999	0.997	0.996
	ResNet50	82.5	0.827	0.942	0.822	0.765	100.0	1.000	1.000	1.000	1.000
	MobileNet-v2	85.0	0.863	0.951	0.852	0.810	97.5	0.975	0.991	0.975	0.967
	GoogleNet	42.5	0.358	0.798	0.313	0.224	95.3	0.953	0.984	0.952	0.937

Acc.: accuracy, Sens.: sensitivity, Spec.: specificity, F1: F1_score.

**Table 10:** Confusion matrix of HS local dataset– ResNet50 (initial results—left, final results—right).

	Ascent	Descent	Walking	Standing		Ascent	Descent	Walking	Standing
**Ascent**	9	2	1	0	**Ascent**	86	0	0	0
**Descent**	2	5	0	0	**Descent**	0	95	0	0
**Walking**	0	2	11	0	**Walking**	0	0	91	0
**Standing**	0	0	0	8	**Standing**	0	0	0	87

**Table 11:** HAR performance metrics in local PD dataset.

		Initial State					Enhanced State				
DL-CNN Epochs: 5 Iteration: 190 Learning rate: 0.001 Batch size: 32	Pre-trained network	Acc. (%)	Sens.	Spec.	F1	MCC	Acc. (%)	Sens.	Spec.	F1	MCC
	ResNet18	94.6	0.949	0.982	0.947	0.929	98.8	0.987	0.996	0.987	0.983
	ResNet50	94.6	0.940	0.981	0.945	0.928	99.0	0.989	0.997	0.990	0.986
	MobileNet-v2	92.9	0.936	0.976	0.931	0.908	99.2	0.992	0.997	0.992	0.989
	GoogleNet	89.3	0.895	0.964	0.896	0.864	97.61	0.973	0.991	0.978	0.975

Acc.: accuracy, Sens.: sensitivity, Spec.: specificity, F1: F1_score.

**Table 12:** Confusion matrix of PD local dataset– MobileNet-v2 (initial results—left, final results—right).

	Ascent	Descent	Walking	Standing		Ascent	Descent	Walking	Standing
**Ascent**	15	1	0	2	**Ascent**	121	1	0	2
**Descent**	1	10	0	0	**Descent**	0	99	0	0
**Walking**	0	0	13	0	**Walking**	0	0	135	0
**Standing**	0	0	0	14	**Standing**	0	0	1	145

**Table 13:** HAR performance in local SS dataset.

		Initial State					Enhanced State				
DL-CNN Epochs: 5 Iteration: 190 Learning rate: 0.001 Batch size: 32	Pre-trained network	Acc. (%)	Sens.	Spec.	F1	MCC	Acc. (%)	Sens.	Spec.	F1	MCC
	ResNet18	74.3	0.690	0.917	0.643	0.591	96.2	0.944	0.987	0.948	0.936
	ResNet50	71.4	0.667	0.903	0.629	0.558	98.1	0.968	0.993	0.973	0.967
	MobileNet-v2	74.3	0.690	0.913	0.650	0.590	97.4	0.960	0.992	0.960	0.952
	GoogleNet	65.7	0.500	0.874	0.563	0.516	79.8	0.655	0.927	0.656	0.647

Acc.: accuracy, Sens.: sensitivity, Spec.: specificity, F1: F1_score.

**Table 14:** Confusion matrix of SS local dataset– ResNet50 (initial results—left, final results—right).

	Ascent	Descent	Walking	Standing		Ascent	Descent	Walking	Standing
**Ascent**	1	1	1	4	**Ascent**	36	0	0	3
**Descent**	0	4	0	1	**Descent**	1	51	0	1
**Walking**	0	0	12	0	**Walking**	0	0	120	0
**Standing**	1	2	0	8	**Standing**	0	1	0	99

**Table 15:** Reference studies with benchmarking datasets.

Study	Method	Augmentation	Accuracy (%)	
			UCI	WISDM
Alawneh et al. [37]	RNN	Moving average and the exponential smoothing	97.9–80.0 *	97.13–83.4 *
Huang et al. [36]	CNN	Step detection based novel augmentation technique- not appropriate for passive activities	-	95.7–86.4 *
Yen et al. [63]	CNN	NA	95.99	-
Jiang and Yin [5]	CNN	NA	97.59	-
Li and Trocan [64]	CNN	NA	95.75	-
Cho and Yoon [65]	CNN	Data sharpening	97.62	-
Proposed framework	CNN	Numerical image conversion + image augmentation	97.0–93.3 *	95.8–86.0 *

* Represents initial results where available.

## Data Availability

Not applicable.
